# Recovery from Exercise in Persons with Myalgic Encephalomyelitis/Chronic Fatigue Syndrome (ME/CFS)

**DOI:** 10.3390/medicina59030571

**Published:** 2023-03-15

**Authors:** Geoffrey E. Moore, Betsy A. Keller, Jared Stevens, Xiangling Mao, Staci R. Stevens, John K. Chia, Susan M. Levine, Carl J. Franconi, Maureen R. Hanson

**Affiliations:** 1 Department of Molecular Biology and Genetics, Cornell University, Ithaca, NY 14853, USA; 2 Department of Exercise Science & Athletic Training, Ithaca College, Ithaca, NY 14850, USA; 3 Workwell Foundation, Ripon, CA 95366, USA; 4 Department of Radiology, Weill Cornell Medical College, New York, NY 10021, USA; 5 EVMED Research, Torrance, CA 90505, USA; 6 Medical Office of Susan Levine, Manhattan, NY 10021, USA

**Keywords:** myalgic encephalomyelitis, chronic fatigue syndrome, 2-day cardiopulmonary exercise test, post-exertional malaise, specific symptom severity, exercise recovery

## Abstract

*Background and Objectives*: Post-exertional malaise (PEM) is the hallmark of myalgic encephalomyelitis/chronic fatigue syndrome (ME/CFS), but there has been little effort to quantitate the duration of PEM symptoms following a known exertional stressor. Using a Symptom Severity Scale (SSS) that includes nine common symptoms of ME/CFS, we sought to characterize the duration and severity of PEM symptoms following two cardiopulmonary exercise tests separated by 24 h (2-day CPET). *Materials and Methods*: Eighty persons with ME/CFS and 64 controls (CTL) underwent a 2-day CPET. ME/CFS subjects met the Canadian Clinical Criteria for diagnosis of ME/CFS; controls were healthy but not participating in regular physical activity. All subjects who met maximal effort criteria on both CPETs were included. SSS scores were obtained at baseline, immediately prior to both CPETs, the day after the second CPET, and every two days after the CPET-1 for 10 days. *Results*: There was a highly significant difference in judged recovery time (ME/CFS = 12.7 ± 1.2 d; CTL = 2.1 ± 0.2 d, mean ± s.e.m., Chi^2^ = 90.1, *p* < 0.0001). The range of ME/CFS patient recovery was 1–64 days, while the range in CTL was 1–10 days; one subject with ME/CFS had not recovered after one year and was not included in the analysis. Less than 10% of subjects with ME/CFS took more than three weeks to recover. There was no difference in recovery time based on the level of pre-test symptoms prior to CPET-1 (F = 1.12, *p* = 0.33). Mean SSS scores at baseline were significantly higher than at pre-CPET-1 (5.70 ± 0.16 vs. 4.02 ± 0.18, *p* < 0.0001). Pharmacokinetic models showed an extremely prolonged decay of the PEM response (Chi^2^ > 22, *p* < 0.0001) to the 2-day CPET. *Conclusions*: ME/CFS subjects took an average of about two weeks to recover from a 2-day CPET, whereas sedentary controls needed only two days. These data quantitate the prolonged recovery time in ME/CFS and improve the ability to obtain well-informed consent prior to doing exercise testing in persons with ME/CFS. Quantitative monitoring of PEM symptoms may provide a method to help manage PEM.

## 1. Introduction

Post-exertional malaise (PEM) is the clinical hallmark of myalgic encephalomyelitis/chronic fatigue syndrome (ME/CFS) and is central to the diagnosis of ME/CFS [1,2]. The nature of PEM is, however, poorly understood, and therefore it is challenging to advise patients with ME/CFS on how to manage their recovery from even mild exertion, such as activities of daily living. For this reason, it is important to better understand the exercise-dose recovery response in persons with ME/CFS.

Prior research has shed some light on exercise-dose recovery response in ME/CFS but has focused more on the physiological response than PEM symptom recovery. The administration of two cardiopulmonary exercise tests (CPETs) separated by 24 h, also known as 2-day CPET, has been used to provide supporting evidence of disability for persons with ME/CFS. The first CPET measures baseline energy-producing capacity and provokes PEM, and the second CPET assesses the ability to reproduce CPET-1 within normal variability. Numerous studies have shown significant alterations in physiological responses to exercise, including alterations in maximal oxygen uptake and the ventilation threshold [3,4,5,6,7]. This inability to reproduce the same physiologic responses after 24 h of recovery is extremely unusual and may be indicative of PEM [6].

PEM is not as objectively measurable as changes in physiological parameters on a 2-day CPET since PEM is a syndrome of subjective symptoms. As part of the 2-day CPET protocol, investigators have commonly obtained free-text narratives about the patient’s symptoms [8]. Unfortunately, this approach is not readily quantifiable or repeatable. Indeed, written narratives from ME/CFS subjects are often quite lengthy and very difficult to characterize. There has been little effort to quantitate PEM symptoms, although one study used visual analog scales (VAS) to assess common ME/CFS symptoms [9].

Since free-living persons with ME/CFS must use symptom ratings to gauge their activity levels, it is important to have subjective tools that persons with ME/CFS can use to better characterize PEM and thereby have a more precise approach to activity pacing so as to avoid exacerbation of PEM. How long does it take a person with ME/CFS to recover following a given dose of exercise? Dose-response is usually examined with a view to estimating the benefits of exercise, but in the ME/CFS community, it is perhaps more important to be able to estimate the risks of exercise [10,11,12].

Our objective in this study was to assess the time course of PEM symptom severity following an exertional stressor (2-day CPET) at a standardized exercise intensity (achieving maximal aerobic capacity on both CPETs), comparing persons with ME/CFS to sedentary but healthy control participants. We sought to follow PEM symptoms until they had returned to the baseline state prior to the 2-day CPET. Key parameters of interest were the time to the peak of PEM symptoms, the change in the severity of PEM symptoms from baseline to peak severity, and the time needed for PEM symptoms to return back to baseline severity.

These characteristics—time to peak effect, the magnitude of peak effect, and rate of decay—are commonly used in pharmacology in order to understand how to properly dose medications. Exercise has been likened to medicine because exercise can promote health and well-being, and indeed exercise training works through the stimulation of endogenous biochemical pathways that are the targets of many modern medications [10]. Like in medications, the goal with dosing exercise should be to avoid both an acute overdose leading to immediate toxicity (i.e., injury or adverse effects) as well as a chronic overdose wherein successive doses are given too frequently and thereby resulting in a gradual rise in baseline symptoms (i.e., overtraining and exhaustion). Therefore, we sought to ascertain if pharmacologic models could be adapted to monitor and better understand PEM.

## 2. Materials and Methods

### 2.1. Subject Selection

This study had three clinical study sites: Ithaca (ITH), Los Angeles (LA) and Weill-Cornell in New York City (WC). All subjects consented to and were screened by a physician—GEM in Ithaca, JC in Los Angeles and SL in New York City. Subjects who agreed to participate in the study were screened to rule out other causes of fatigue, including a medical history review for affective disorders, neuro-inflammatory disorders, musculoskeletal disorders, fibromyalgia, cardiometabolic disease, endocrinological conditions such as thyroid disorders and diabetes. A complete review of systems was obtained, with added emphasis on symptoms characteristic of ME/CFS. A physical exam was performed to screen for conditions associated with fatigue, including cardiovascular, pulmonary, neuromuscular and skeletal conditions such as heart failure, cardiovascular disease, obstructive or restrictive pulmonary disease, hyper- or hypothyroidism, motor and sensory abnormalities, arthritis and undiagnosed malignancies. Blood samples for complete metabolic profile, complete blood count, T4 level and A1c level were obtained and analyzed by Quest Diagnostics Incorporated. Urine was screened for cannabis, narcotics, and psychoactive medications. Female subjects had a pregnancy test.

To be included in the study, subjects had to be 18–70 years of age, not regularly participate in any form of exercise, and have normal blood tests and urine free of any substances. Females had a negative pregnancy test. Subjects who had any clinically significant abnormality in the blood tests or who had a co-morbid condition associated with fatigue, including diabetes, were excluded. Subjects were assigned to the ME/CFS group if their medical history satisfied the Canadian Clinical Criteria (CCC) for diagnosis of ME/CFS [1]. Subjects were assigned to the control (CTL) group if their medical history did not meet the CCC criteria for ME/CFS. We specifically recruited people who were generally healthy but chronically sedentary to serve as the control group and sought to age- and gender-match controls to subjects in the ME/CFS group.

### 2.2. Data Collection and Resolution

Subjects who met the study inclusion criteria (92 ME, 81 CTL) underwent the 2-day CPET protocol, with both exercise tests performed 24 h apart in mid-morning. Maximal oxygen uptake (VO2_max_) was confirmed in each test by the presence of two of three criteria: (1) a heart rate greater than 85% of the age-predicted maximum, (2) a respiratory exchange ratio of greater than 1.10, and (3) a high rating of perceived exertion (≥17 on the 6–20 point RPE scale) indicative of volitional exhaustion. Subjects who failed to achieve VO2 max on both CPETs were excluded.

**Figure 1:**
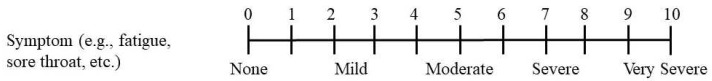
Visual Analogue/Likert Scores of Symptoms.

To quantitate PEM, we chose the Specific Symptom Severity questionnaire (SSS) [9]. This questionnaire has nine domains, using a combination of 10-point Likert and visual analog scales for each domain. The nine domains are fatigue, brain fog, sore throat, tender lymph nodes, myalgia, arthralgia, headache, disturbed sleep and PEM. In our system, the visual analog score and the Likert scores were aligned, as shown in Figure 1.

We also solicited free-text input, as in prior studies. SSS scores were obtained immediately prior to both CPETs, 24 h after the second CPET, and then every two days for ten days after the first CPET. A number of ME/CFS subjects completed additional SSS forms beyond ten days until they felt recovered. A baseline SSS was also obtained as part of a battery of questionnaires (not reported here) a few weeks prior to the first CPET.

When the subjects’ records had been returned to the study sites, one investigator (GEM) reviewed the SSS forms to corroborate each subject’s SSS scores with that subject’s narrative and stated an estimate of their recovery. Particularly for ME/CFS subjects, the time it took SSS scores to return to the pre-CPET level and the stated recovery time in the written narratives were often not in agreement. This was deemed not to be due to one being correct and the other incorrect but rather to the difficulty of defining and sensing full recovery and to slightly different notions arising from using two different methods. All the SSS scores were reviewed, and a recovery time was estimated for how long it took for all the scores to return to the pre-CPET1 scores. This judged recovery time estimate was compared with what the subject stated in their narrative, and a final estimate was established as a “judged recovery time”. We did not track what the investigator thought versus what the subject thought but rather were attempting to resolve conflicting responses between the subject’s own SSS scores and narrative. Controls who stated that they recovered in less than a day were assigned one day.

In some cases, our recovery time estimate did not concur with the subject’s stated recovery time, and rarely the subject stated that they had not recovered. In such cases, we attempted to contact the subject to get greater clarity on how long they felt that it took to recover. Most subjects we attempted to contact replied, but after returning the SSS forms, they had officially completed their responsibilities to the study and were under no obligation to respond. The subjects from LA and NYC never had prior contact with the investigator calling them (GEM), which may have affected their willingness to respond. Indeterminant cases where we were unable to determine a judged recovery time were excluded from the analysis.

Study data were collected on paper and entered using dual-entry methods into a REDCap shared library hosted by Weill-Cornell Medicine [13,14,15].

### 2.3. Data Analysis

All analyses, with correction for repeated measures when appropriate, were performed using JMP Pro 16 (SAS, Cary, NC, USA). Bivariate non-parametric analyses were performed between groups, and curve-fitting was performed with lambda = 1.0. Power analysis was not performed, as the study size was determined by a much larger project to which this study was added.

JMP 16 Pro has built-in curve-fitting for three pharmacokinetic models for estimating the level of a drug in the body: oral dose one-compartment (i.e., water), intravenous dose two-compartment (i.e., water and lipid), and a bi-exponential four-parameter model. Because our methods seemed less comparable to a rapid-onset intravenous administration, we applied our data to the oral dose one-compartment and to the bi-exponential four-parameter models.

The way a pharmacokinetic model would be tested with medication is as follows: a dose would be administered, and then blood levels of the drug would be measured at subsequent time intervals. Thus, for these analyses, the mean SSS score (of all nine PEM domains) was equated to a blood level of a drug. In other words, the mean SSS score would be modeling some biological phenomenon mediating PEM as if it were a measurable compound in the subject’s blood. This is plausible because exercise affects biochemistry, so exercise can be reasonably expected to alter the kinetics of circulating compounds [16]. Therefore, pharmacokinetic models can be reasonably expected to associate with side effects of exercise, such as soreness and fatigue.

To make such comparisons, however, our data require adapting to pharmacokinetic models because of assumptions underlying the models. Since all study subjects were sedentary prior to the first CPET, and if exercise is the “drug”, there theoretically could not be a drug level attributable to the CPETs prior to performing the first CPET. The drug level attributable to the CPETs would be 0, but our mean SSS scores were not 0. Thus, the mean SSS (*MeanSSS*) scores were first normalized by subtracting the pre-CPET1 from all SSS values so that the pre-CPET score was 0, forcing the model to look at the change in *MeanSSS*. The equation in JMP for the oral dose one-compartment model was:

$$MeanSSS=\left(\left(a\cdot b\cdot c\right)/\left(c-b\right)\right)\cdot \left(Exp\left(-b\cdot SurveyDay\right)-Exp\left(-c\cdot SurveyDay\right)\right)$$

where *a*, *b*, and *c* are coefficients where the computer solves for the value of *a*, *b*, and *c* and then tests the fit of the curve. These coefficients reflect complex integrated physiological processes not even well-understood by pharmacologists, and for our purposes, the biology underlying these coefficients is not important. In this model, subtracting out the mean SSS score from each day resulted in some *MeanSSS* scores < 0, which were ignored since there cannot be a negative drug level. Moreover, scores lower than baseline, feeling better than before the first CPET, would theoretically represent full recovery from the exercise perturbation.

The four-parameter bi-exponential pharmacokinetic model solves for four coefficients (*a*, *b*, *c*, *d*) in the following equation:

$$MeanSSS=a\cdot Exp\left(-b\cdot SurveyDay\right)+c\cdot Exp\left(-d\cdot SurveyDay\right)$$

Again, the nature of the coefficients is not important. For this model, the pre-CPET1 values of mean SSS scores were normalized to 0, but rather than negating all subsequent *MeanSSS* scores < 0, a constant of 2.45 was added to all SSS values so that all *MeanSSS* scores were ≥ 0. This was necessary because the model would not function unless all *MeanSSS* values were ≥ 0. The value of this constant has no meaningful significance.

It is important to understand that the objective of testing these models was mainly to see if there is a quantitative way to estimate the peak as well as the rate of recovery of PEM symptoms after a standardized dose of exercise. At present, persons with ME/CFS do not have a reliable method of understanding how much a dose of activity is going to provoke PEM, nor how long the PEM will last. There is an unmet need in the research literature to help people understand how physical activity impacts the symptoms of ME/CFS.

## 3. Results

The present study was part of a larger study to investigate molecular mechanisms of ME/CFS. Detailed exercise test results of the 2-day CPET protocol will be reported separately. For the present analysis, there is a smaller sample size (ME/CFS *n* = 80, CTL *n* = 64) than the total number of CPET participants (92 ME/CFS, 81 CTL) due to missing data (SSS forms either not returned or completed) and to subjects who did not meet two of the three criteria for reaching VO2max on both CPETs. The ME/CFS group had an outlier in judged recovery time who did not feel recovered after one year and an individual whose recovery data were incomplete, resulting in a final sample size of ME *n* = 78, CTL *n* = 64.

Demographic characteristics of the study population and our main findings are shown in Table 1. Female ME and CTL had similar ages (F = 3.07, *p* = 0.08), as did male ME and CTL (F = 0.13, *p* = 0.72). Subjects were instructed to rest prior to their 2-day CPET studies. Examining the baseline SSS scores to the pre-CPET1 scores, a prominent finding was that ME subjects were substantially more fatigued at baseline in their normal day-to-day lives than at the pre-test assessment prior to CPET1. Mean SSS scores—the mean of a subject’s SSS scores for all nine domains—at baseline were significantly higher than at pre-CPET1 (5.70 ± 0.16 vs. 4.02 ± 0.18, *p* < 0.0001).

There was a highly significant difference in judged recovery time between ME/CFS and CTL (see Table 1), but judged recovery times for females and males were similar in the ME/CFS group (Chi^2^ = 0.31, *p* = 0.58) and in the CTL group (Chi^2^ = 1.30, *p* = 0.25). The range of ME/CFS patient recovery was 1–64 days, and the range in CTL was 1–10 days.

The number of years that the subjects reported they had been ill with ME/CFS had no effect on judged recovery time (F ratio = 0.76, *p* = 0.47, see Table 2), indicating that duration of illness was not an important factor.

**Figure 2:**
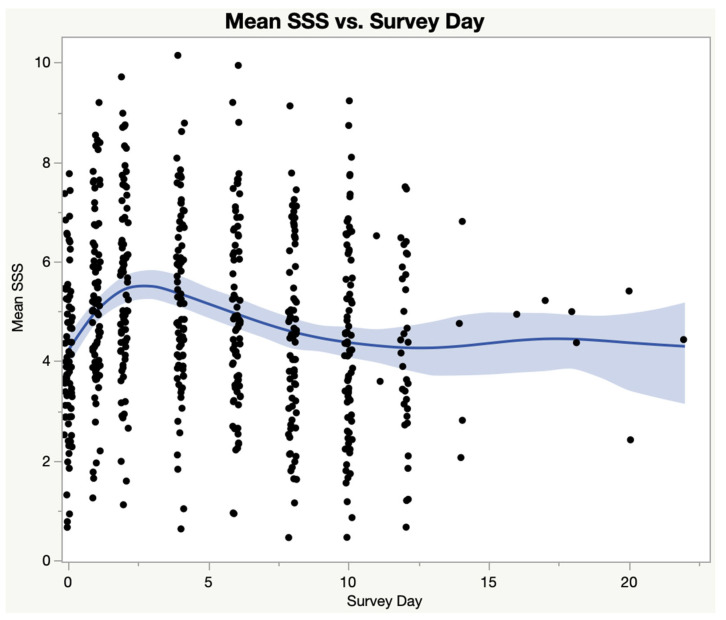
Mean score of all nine domains of the SSS instrument—ME/CFS subjects only. The shaded area represents the 95% confidence interval. Note that there are a significant number of data points beyond day 10, representing about 7–8% of ME/CFS subjects. Each dot is the mean SSS score for an individual ME/CFS subject; Blue line is the spline curve representing the average of all data points in the figure; Light blue area is the 95% confidence interval.

Figure 2 shows a graph of mean SSS scores—the mean score of all nine domains in each SSS survey—in the ME group by Survey Day. SSS scores that were submitted beyond 10 days are shown in Figure 2.

**Figure 3:**
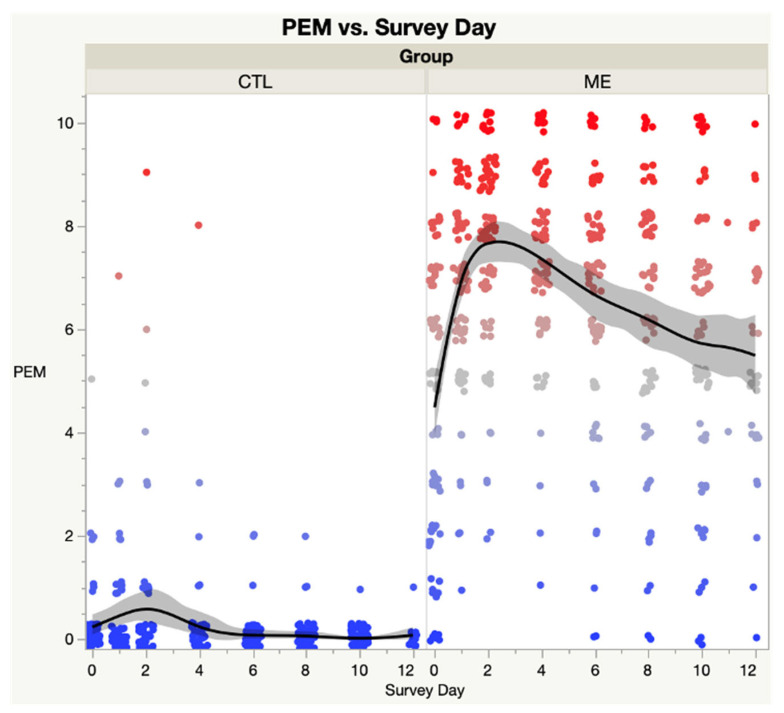
Post-Exertional Malaise (PEM) domain score of SSS instrument—ME/CFS vs. CTL. The shaded area represents the 95% confidence interval. Each dot represents the PEM score for a single subject; Blue dots are low scores, red dots are high scores; Black lines represent the spline curves for group averages; Grey shaded areas are the 95% confidence intervals.

Figure 3 contrasts the spline curves and confidence intervals for ME vs. CTL groups in PEM by Survey Day. ANOVA reveals significant differences between groups (F = 2555.8, *p* < 0.0001) and by survey day (F = 5.37, *p* < 0.05).

**Figure 4:**
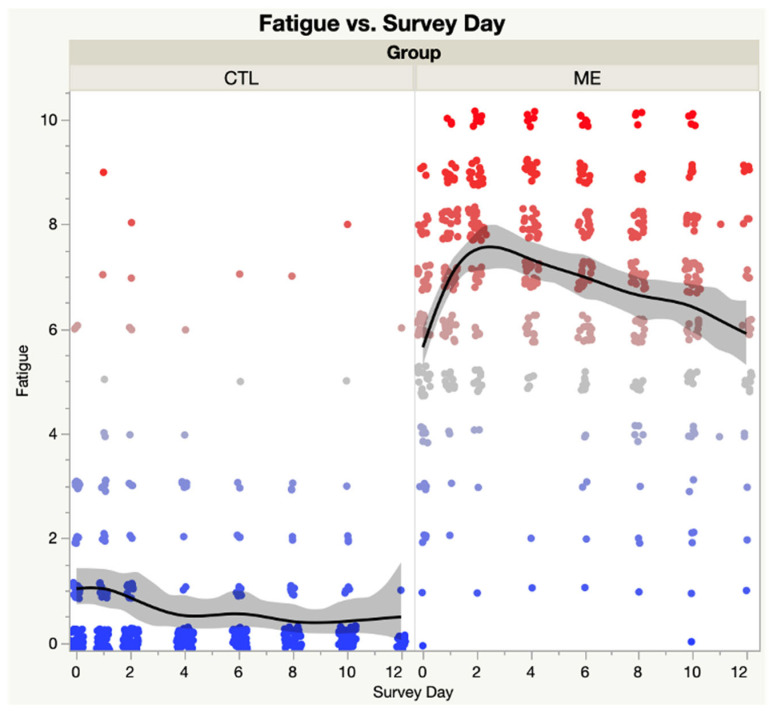
Fatigue domain score of SSS instrument—ME/CFS vs. CTL. Shaded area represents the 95% confidence interval. Each dot represents the PEM score for a single subject; Blue dots are low scores, red dots are high scores; Black lines represent the spline curves for group averages; Grey shaded areas are the 95% confidence intervals.

Figure 4 contrasts the spline curves and confidence intervals for ME vs. CTL groups in Fatigue by Survey Day. ANOVA revealed significant differences between groups (F = 3180.5, *p* < 0.0001) and by survey day (F = 7.87, *p* < 0.01).

**Figure 5:**
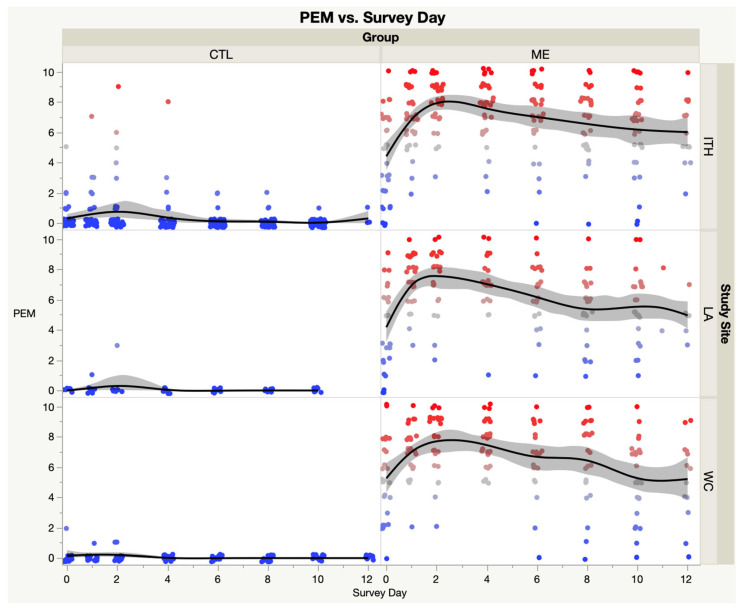
PEM domain score of SSS instrument by study site—ME/CFS vs. CTL. Fatigue domains score of SSS instrument—ME/CFS vs. CTL. Each dot represents the PEM score for a single subject; Blue dots are low scores, red dots are high scores; Black lines represent the spline curves for group averages; Grey shaded areas are the 95% confidence intervals.

Figure 5 compares the three study sites for the ME group in PEM by Survey Day. ANOVA revealed that the ME groups were similar between all three sites (F = 2.51, *p* = 0.09).

**Figure 6:**
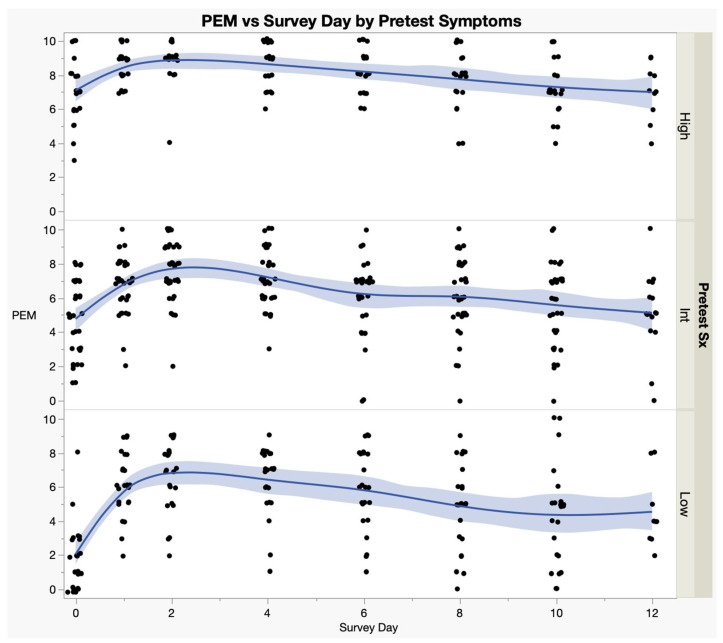
Post-Exertional Malaise (PEM) Recovery by Level of Baseline Symptoms. PEM domain scores of the SSS instrument by the severity of pre-test symptoms: High, Intermediate (Int) or Low—ME/CFS only. Each dot is the mean PEM score for an individual ME/CFS subject; Blue lines are the spline curves representing the average of all data points in each subgroup; Light blue areas are the 95% confidence intervals.

Figure 6 shows the time courses of PEM in ME subjects by whether they had low, high or medium symptoms at baseline. The low, medium and high rating was based on the mean SSS of all nine domain scores. High was defined as the top quartile, low was defined as the bottom quartile, and medium group was the middle two quartiles. There were significant differences in PEM scores between groups (F = 66.4, *p* < 0.0001), but there was no difference in recovery between the groups (F = 1.12, *p* = 0.33).

**Figure 7:**
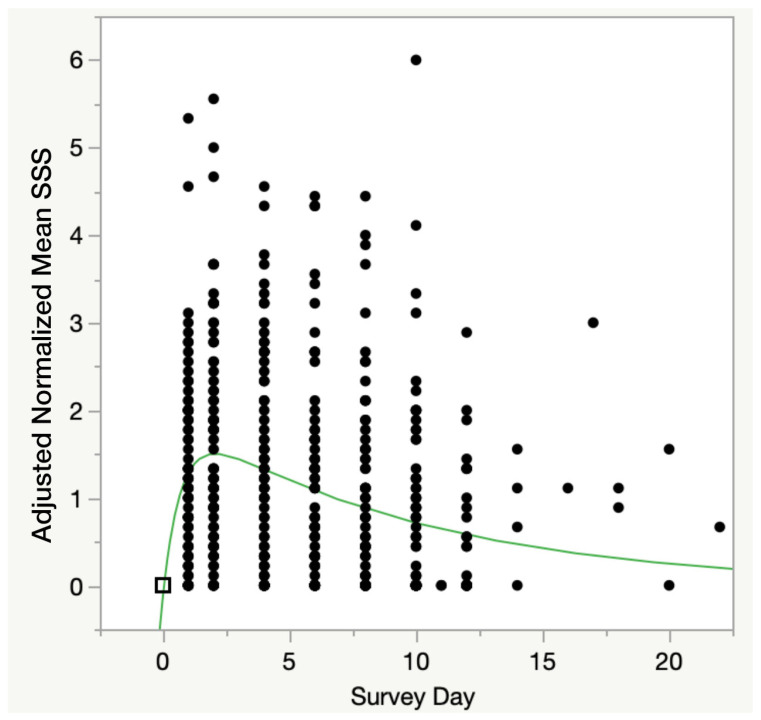
Oral Dose Pharmacokinetic Model of PEM Response to 2-day CPET. Oral dose pharmacokinetic model of mean SSS score. Pre-test scores were normalized to 0, and on subsequent survey days, all mean SSS scores ≤ 0 were set to 0 as representing full recovery from the 2-day CPET. F ratio = 8.41, *p* < 0.0005. Black box represents that in this model all subject’s Mean SSS scores were normalized (set to 0 at time 0, or pre-CPET1, for all subjects); Black dots are all other normalized Mean SSS scores for each subject, at subsequent days; Green line is the mathematical solution for the model.

Figure 7 shows the oral dose one-compartment pharmacokinetic modeling showed a highly significant relationship for all three parameters in the model ((a): Chi^2^ = 66.7, *p* < 0.0001; (b): Chi^2^ = 22.9, *p* < 0.0001; (c): Chi^2^ = 51.3, *p* = 0.001). This model also calculated that peak symptoms were approximately 1.5 units higher than immediately prior to CPET1 and occurred 24 h after CPET2. In contrast to the four-parameter model, the decay rate was slightly slower at 0.10 ± 0.02 units per day and did not return to the pre-CPET1 value until after three weeks. This difference is due to treating all values of mean SSS < pre-CPET1 mean SSS as being recovered.

**Figure 8:**
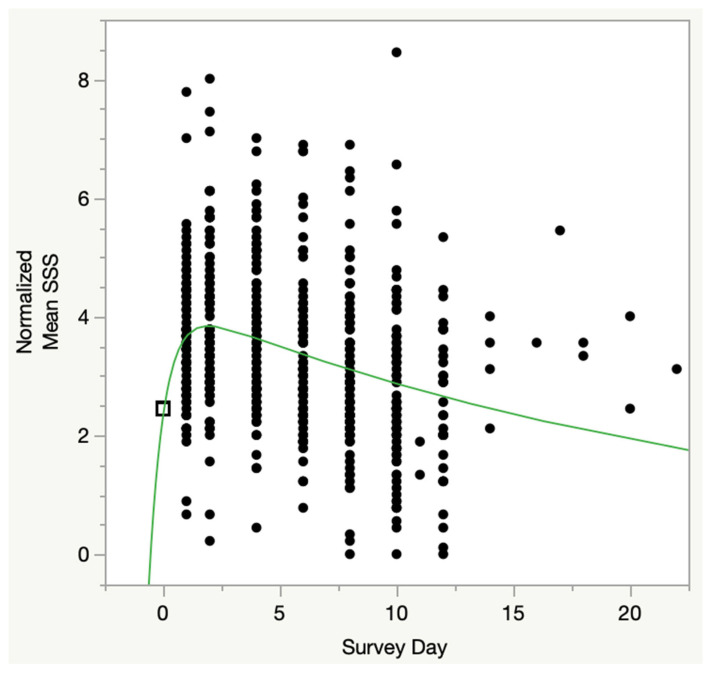
Four Parameter Pharmacokinetic Model of PEM Response to 2-day CPET. Four-parameter pharmacokinetic model of normalized mean SSS scores. Scores were adjusted for pre-test symptoms, such that all pre-test values were 2.45 (open box at day 0). Black box represents that in this model all subject’s Mean SSS scores were normalized (set to 2.45 at time 0, or pre-CPET1, for all subjects); Black dots are all other normalized Mean SSS scores for each subject, at subsequent days; Green line is the mathematical solution for the model.

Figure 8 shows the four-parameter pharmacokinetic modeling showed a highly significant relationship for all four parameters in the model ((a): Chi^2^ = 388.1, *p* < 0.0001; (b): Chi^2^ = 32.8, *p* < 0.0001; (c): Chi^2^ = 51.3, *p* < 0.0001; (d): Chi^2^ = 7.91, *p* < 0.005). The model calculated that peak symptoms were, on average, approximately 1.5 units higher than immediately prior to CPET1, occurred about 24 h after CPET2, and returned to the pre-CPET1 value at about two weeks. Comparing Figure 7 to Figure 8, the main differences are that the Oral Dose (Figure 7) model assumes no exercise-related effects prior to the CPETs (Mean SSS = 0), whereas Figure 8 shows exercise-related effects prior to the CPETs (Mean SSS > 0). Both are normalized, wherein all subjects are given equal scores immediately prior to CPET1. 

**Figure 9:**
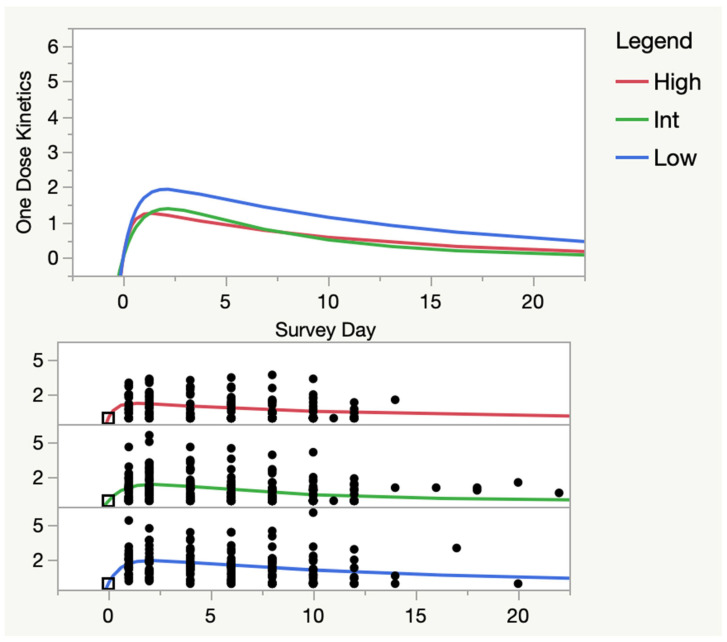
One-Dose Pharmacokinetic Model by Pre-CPET 1 Symptoms. One dose pharmacokinetic model by pre-CPET1 symptoms—ME/CFS only. High, Intermediate (Int) and Low groups were assigned by their pre-CPET1 mean SSS scores; High and low were the highest and lowest quartiles, Int was the middle two quartiles. Intermediate and high groups were not significantly different from the low group: High group vs. Int, F ratio = 0.05, N.S.; Low, F ratio = 0.01, N.S.; Int group vs. Low: F ratio = −0.04, N.S. Black boxes represent that in this model all subject’s Mean SSS scores were normalized (set to 0 at time 0, or pre-CPET1, for all subjects); Black dots are all other normalized Mean SSS scores for each subject, at subsequent days; Red, green and blue lines are the mathematical solutions for each group in the model.

Figure 9 shows the one-compartment pharmacokinetics of mean SSS by high, intermediate and low pre-CPET1 symptom groups. JMP Pro 16 does not have the ability to run statistics between pharmacokinetic models. These results suggested a higher area under the curve (AUC), i.e., more symptoms, for the low symptom group (Low = 31.0 ± 6.9 vs. High = 15.2 ± 4.4 and Int = 12.9 ± 2.1; mean ± s.e.m.). However, the “elimination rates” appeared to be similar for all three groups (Low = 0.073 ± 0.024, High = 0.094 ± 0.042, Int = 0.149 ± 0.050; mean ± s.e.m.), as were the times to peak response (Low = 2.11 ± 0.53 d, High = 1.35 ± 1.13 d, Int = 2.20 ± 0.53 d; mean ± s.e.m.). The one-compartment pharmacokinetic model reinforced the visual impression that the peak response in symptoms may be blunted in the high symptom group (Low = 1.94 ± 0.30, Int = 1.39 ± 0.52, High = 1.26 ± 0.23; mean ± s.e.m.).

## 4. Discussion

The present data provide a standardized quantitative estimate of recovery response in persons with ME/CFS from a well-characterized dose of exercise. Compared to sedentary controls, persons with ME/CFS had a substantially longer exacerbation of symptoms. Multiple analyses showed that, on average, subjects with ME took about two weeks to recover from the 2-day CPET. In contrast, sedentary controls recovered in two days, with many subjects saying they had recovered in one day and some even claiming the same day in their narrative reports.

The current findings show that the 2-day CPET is a highly sensitive method for supporting the diagnosis of ME/CFS, though our study did not examine specificity for ME/CFS. In our collective experience with exercise testing, however, we are not aware of any other condition prior to 2020, including Gulf War Illness, that exhibited this phenomenon [17]. Thus, while not pathognomonic for ME/CFS, a markedly prolonged recovery from the 2-day CPET is very highly suggestive of the diagnosis. A number of individuals who have had COVID-19 since January 2020 are now reporting PEM, one of a multitude of symptoms that evidently can occur from post-SARS COV2 illness [18].

Banister and colleagues used mathematical modeling to examine the fitness and fatigue responses to exercise training and particularly to running [19]. These investigators further attempted to model fatigue by linking physical fatigue to serum enzymes (e.g., lactate dehydrogenase, creatine kinase) [20]. In their model, a “dose” of the exercise was called a “Trimp” or *training impulse*, which resulted in an immediate decline in performance due to fatigue, followed by an adaptive response in fitness. In this model, performance was a combination of fitness and fatigue, which were modeled as exponential functions (fatigue decaying and fitness increasing). The cumulative effect of successive Trimps yields an increase in performance on the presumption that the timing of successive Trimps occurs *after* the individual has recovered from fatigue. These early efforts in modeling physical performance did not venture into exhaustion phenomena, but the model would predict that superposition of successive Trimps on an individual who was insufficiently recovered and thus continuing to suffer from fatigue while not yet achieving an adaptation in fitness would, over time, yield a gradual increase in fatigue such as one sees in exhausted athletes suffering from overtraining. Overtraining is a phenomenon of too much physical stress without enough recovery, leading to decompensation and a reduction in performance rather than adaptation and an increase in performance.

The exponential modeling of fitness and fatigue used by Banister and colleagues is very similar to the pharmacokinetic modeling of drug levels. Thus, we were curious to see if simple pharmacokinetic models could be applied to SSS scores. If pharmacokinetic modeling tracks subjective PEM symptoms, then one might be able to use such modeling to manage physical activity, much in the way that pharmacologists model blood levels of toxic medications to ensure that the medication falls to a low level before giving the patient a subsequent dose. The success of our pharmacokinetic models in tracking SSS scores at very high significance shows that the decay rate of fatigue and PEM symptoms is *extremely prolonged* in persons with ME/CFS. Thus, in persons with ME/CFS who do not allow for several days of recovery after physical stress, albeit at a low absolute level of fitness, there is a high risk of overtraining phenomena.

As a phenotype, persons with ME/CFS respond to two bouts of brief high-intensity exercise with prolonged exhaustion, as if they are overtrained. The phenomenon of being deconditioned yet overtrained is supported by the striking observation that, at baseline in their home environment, our ME subjects reported significantly higher symptoms on the SSS than they did prior to the first CPET. Using the quartile thresholds for the pre-CPET1 SSS survey (as in Figure 5), 52 of 80 subjects–*fully two-thirds*–exceeded the threshold to be in the “High” symptom group at their baseline survey. Twenty-seven of 80 were in the “Mid” pre-CPET1 symptom category (vs. 36 at the pre-CPET1 survey), and *only one of 80* was in the “Low” pre-CPET1 symptom category (vs. 24 at pre-CPET1).

We advised subjects to rest in the days prior to the 2-day CPET protocol, largely so that the ME subjects would not arrive at the study site already exhausted. While we do not believe any of our subjects typically exert themselves at home as vigorously as we had them do during the CPETs, it is likely that persons with ME constantly live in the long tail of the recovery response. While activities of daily living are not as stressful as the 2-day CPET, recovery from less intense activities of daily living is likely to follow a similar decay curve. Such a response to physical activity would be consistent with the ubiquitous complaint from persons with ME that they have constant and persistent PEM. Most persons with ME would constantly experience exertion falling on an incompletely recovered decay curve, and thus their symptoms would increase to a high steady-state level.

Judged recovery time was not affected by the level of baseline symptoms. Our clinical team had expected that persons who had higher SSS scores prior to CPET1 would have more severe symptoms and more prolonged recovery. The data, however, shows that recovery is not affected by the severity of symptoms prior to vigorous activity and that—at least when using a VAS/Likert design—there may be a ceiling effect on the subjective severity of symptoms.

On social media, some patients have posted that they experienced a very prolonged recovery from the 2-day CPET. Given the potential for prolonged and potentially severe disability in ME/CFS, these anecdotes have prompted hesitancy to undergo a 2-day CPET. As a response to such patient advocacy, we monitored recovering subjects in provocation studies that were primarily designed to look for molecular mechanisms of PEM. Prior to asking a person with ME/CFS to undergo a 2-day CPET, it is important to receive informed consent, and the most significant risk for a person with ME/CFS is that she or he will have a significantly prolonged and disabling recovery. Our data suggests that around 7–8% will have a prolonged recovery of 1–2 months, with a very small percentage of ME subjects feeling that they never recover.

It is difficult to verify a participant’s perception that a 2-day CPET leads to non-recovery because once the subjects left our laboratories after the 2nd CPET, they returned to their own environments, and we had no control over internal and external stressors. It is thus not possible to conclude that the 2-day CPET itself was the sole proximate cause of non-recovery, though we acknowledge that it could seem that way to any subject who does not recover.

One ME outlier was excluded for non-recovery; this subject was in the low-symptom group prior to the 2-day CPET. We had several phone conversations with this subject over the course of one year, after which he reported that he did not feel like he had ever recovered. This subject tried a number of treatments, all of which he stated were unsuccessful. We stopped following him after one year. He asserted that his ME/CFS was improving to a point where he was feeling optimistic about having a more normal life and expressed great surprise with his non-recovery.

There are a number of design weaknesses in this study. Foremost, the present protocol was added as a safety measure for a larger project that was not designed to study recovery as a primary objective. As a result, the administration of the SSS scoring sheets was not optimal for objectivity. Subjects were provided with SSS sheets for days 2, 4, 6, 8 and 10, as well as one extra sheet. After day 10 of recovery, subjects were instructed to mail the SSS sheets back to the study sites. However, if they were still symptomatic, they were asked to continue completing SSS forms until they felt recovered. Subjects thus had each of the prior SSS sheets and could have looked back to compare scores from prior days. A more robust protocol would have been the electronic submission of the scores, wherein the subjects would only indicate how they felt that day and not be able to compare their responses on prior days. By providing 10 SSS forms and one extra, we may have subliminally suggested that it was going to take about 10 days to recover. With the electronic submission of SSS scores, study site computers could have calculated in real time when the scores had returned to baseline.

The procedure of returning the SSS sheets after subjects had completed the full protocol induced delays in assessing recovery, particularly in persons who took longer than 10 days. We had a small number of excluded subjects for whom we had incomplete data and could not generate a judged recovery time, though we know it took longer than 10 days. Given that we were primarily interested in the tail of the recovery curve, using a last-observation-carried-forward analysis does not fit the situation. In all, a very large majority of subjects provided complete records, so while it is probable that the true recovery time is longer than our results (12.7 days), the error is probably not large.

Many of the Ithaca subjects had to travel, which may have affected their recovery, though the data show that the Ithaca cohort recovered similarly to the NYC and LA cohorts. Also, autonomic and neuroendocrine (especially gastro-intestinal) symptoms are not elements of the SSS scoring and thus were not followed. At least one subject ascribed to delayed recovery that she noted was due to prolonged gastro-intestinal discomfort and not captured on the SSS.

Another weakness was that subjects were free-roaming between and after CPETs, so their physical activity outside of the CPETs was not controlled. Subjects were advised that fluid and electrolyte supplements might be beneficial but were on their own in choosing to use them. Indeed, there were very many subjects with ME/CFS and a few CTL subjects whose SSS scores peaked, declined and then went back up again. In such individuals, it is very difficult to ascertain whether or not there is truly a bi-phasic peak in SSS scores in response to a 2-day CPET stimulus. Other patterns we occasionally observed were what appeared to be a delayed onset of PEM symptoms, with symptoms not increasing until days after the 2-day CPET. Such phenomena are difficult to explain with the known acute physiologic responses to acute exercise. One issue could well be that subjects feel recovered, but are not, and thus increase their activities and unwittingly provoke a worsening of symptoms. In addition, external variables beyond our control could increase stress and bring about such symptoms. Controlling rest more rigorously after a 2-day CPET would be difficult and costly. Thus, while there were unquestionably subjects whose SSS scores were higher several days after the 2-day CPET, we conclude that these response curves were most likely noise and variability in external stimuli. Neither the spline curves nor either of the pharmacokinetic models shows even a trace of bi-phasic behavior. If there are such persons, it seems likely that they are reflecting more external variables and personal behaviors rather than normative traits of persons with ME/CFS.

## 5. Conclusions

ME/CFS subjects took an average of almost two weeks to recover from a 2-day CPET, whereas sedentary controls only needed an average of two days. Almost 10% of subjects with ME/CFS took more than three weeks to recover, with one subject (~1%) with ME/CFS who felt he had not recovered after a full year. Recovery time back to the pre-CPET1 level was not affected by the severity of symptoms prior to the 2-day CPET. These data improve the ability to obtain well-informed consent prior to doing exercise testing as an element of establishing disability in persons with ME/CFS.

More important, our pharmacokinetic model findings should be viewed with great interest by clinicians who manage ME/CFS as well as patients and advocates for persons with ME/CFS. Our study is the first attempt to rigorously examine the timeline of recovery from an exertional stressor in persons with ME/CFS. If a person with ME/CFS were to take standard advice on exercise and do multiple days of exercise each week, our pharmacokinetic models suggest that they would superimpose additional peak responses on top of a recovery curve that would still be nearly at its peak. Our data suggest that graded exercise therapy almost certainly would cause harm. Small wonder, therefore, that graded exercise therapy has fallen into disfavor in the ME/CFS community. More research needs to be done to help clarify the utility of rigorous symptom tracking in the management of PEM. Until such data become available, clinicians, patients and advocates alike should be aware of the extremely prolonged time required of persons with ME/CFS–male or female–to recover following an exertional stressor.

In summary, the present data suggest that the “half-life” of recovery—the time it takes for PEM symptoms to diminish by one-half—from maximal aerobic exercise in a sedentary control is a few hours, but that in a person with ME/CFS, the half-life is a few *days*. These findings provide robust support for those who voice caution against using graded exercise therapy in persons with ME/CFS.

## Figures and Tables

**Table 1:** Judged Recovery Time by Demographic Group.

	ME Female (Mean ± s.e.m)	CTL Female (Mean ± s.e.m)	ME Male (Mean ± s.e.m)	CTL Male (Mean ± s.e.m)
Sample	*n* = 60	*n* = 48	*n* = 18	*n* = 16
Age	46.7 ± 1.5 years	42.5 ± 2.0 years	43.2 ± 2.5 years	44.6 ± 3.1 years
Judged Recovery	12.7 ± 1.1 days *	2.1 ± 1.2 days	12.5 ± 1.3 days **	1.9 ± 1.3 days

*—Chi^2^ = 67.5, *p* < 0.0001 compared to female controls; **—Chi^2^ = 22.8, *p* < 0.0001 compared to male controls. ME = ME/CFS, CTL = control

**Table 2:** Recovery Time by Duration of ME/CFS.

Duration of ME/CFS (Years)	Judged Recovery Time (Mean ± s.e.m)
0–3	14.9 ± 2.7
4–9	10.9 ±1.9
>9	13.0 ± 2.0

Recovery time was not significantly affected by the duration of ME/CFS.

## Data Availability

The data presented in this study are in the supplementary files and will also be available at MapMECFS (https://www.mapmecfs.org/, accessed on 10 February 2023).

## Supplementary Materials

The following supporting information can be downloaded at: https://www.mdpi.com/article/10.3390/medicina59030571/s1.
